# Residual matrix from different separation techniques impacts exosome biological activity

**DOI:** 10.1038/srep23550

**Published:** 2016-03-24

**Authors:** Lucia Paolini, Andrea Zendrini, Giuseppe Di Noto, Sara Busatto, Elisabetta Lottini, Annalisa Radeghieri, Alessandra Dossi, Andrea Caneschi, Doris Ricotta, Paolo Bergese

**Affiliations:** 1 Department of Molecular and Translational Medicine and INSTM, University of Brescia, Brescia, Italy; 2 Department of Chemistry and INSTM, Laboratory of Molecular Magnetism, University of Firenze, Sesto Fiorentino (Firenze), Italy

## Abstract

Exosomes are gaining a prominent role in research due to their intriguing biology and several therapeutic opportunities. However, their accurate purification from body fluids and detailed physicochemical characterization remain open issues. We isolated exosomes from serum of patients with Multiple Myeloma by four of the most popular purification methods and assessed the presence of residual contaminants in the preparations through an *ad hoc* combination of biochemical and biophysical techniques - including Western Blot, colloidal nanoplasmonics, atomic force microscopy (AFM) and scanning helium ion microscopy (HIM). The preparations obtained by iodixanol and sucrose gradients were highly pure. To the contrary, those achieved with limited processing (serial centrifugation or one step precipitation kit) resulted contaminated by a residual matrix, embedding the exosomes. The contaminated preparations showed lower ability to induce NfkB nuclear translocation in endothelial cells with respect to the pure ones, probably because the matrix prevents the interaction and fusion of the exosomes with the cell membrane. These findings suggest that exosome preparation purity must be carefully assessed since it may interfere with exosome biological activity. Contaminants can be reliably probed only by an integrated characterization approach aimed at both the molecular and the colloidal length scales.

Exosomes are gaining ever-increasing attention due to their intriguing biology and emerging therapeutic opportunities^1^,^2^,^3^,^4^. Exosomes are vesicles with a size of 50–100 nm, which are secreted by cells into the extracellular space and play an important role in cell communication as cargoes of several specific proteins and RNAs. For example, exosomes are to date considered playing a pivotal role in information transfer in hematological malignancies^5^. On the other hand, being contained in most body fluids (including saliva^6^, plasma^7^, urine^8^, amniotic fluid^9^) they promise to be an effective mean to fluid biopsy. Reliable separation and detailed characterization of extracellular vesicles are mandatory steps to advance biological understanding and biotechnological exploitation of exosomes which still need to be addressed^10^,^11^.

A variety of techniques for exosome separation is flourishing, including ultracentrifugation, density gradient, filtration, microfluidics techniques and precipitation kits^12^,^13^,^14^,^15^. In addition, in the last few years, micro- and nanodevice based isolation techniques (nanowired-on-microcapillary trapping, acoustic sorting and immunoaffinity-based isolation) are being tested^16^. However, key analytical parameters, as separation yield or preparation purity, remain open issues^11^,^17^,^18^.

Autoimmune diseases, hematologic disorders, infections, and cancer associated with elevated exosomes counts are also characterized by accelerated formation of immune and protein complexes^11^. These aggregates share several biophysical parameters with exosomes - such as size, surface charge and light absorption - which may strongly affect vesicles purification^11^,^17^,^19^. Protein contamination can also alter or even invalidate proteomic and transcriptomic studies on exosomal proteins and genetic material^20^,^21^,^22^,^23^.

In this article we investigate the effective ability of the most popular protocols to separate exosomes from contaminant single/aggregated proteins and lipids and we analyze the effects of eventual residual contaminants on the biological activity of the preparations. To achieve this goal we take advantage of an original combination of classical bioanalytical methods, colloidal scale (viz. nanoscale) characterization techniques and *in vitro* assays. Exosomes were isolated from a pool of sera from patients affected by Multiple Myeloma (MM), by using four different isolation protocols: serial centrifugation steps (P3), iodixanol or sucrose density gradient and a commercial kit based on vesicles precipitation (Exo PK). The protein overall content of the preparations was quantified by Bradford assay and samples were analyzed by Western Blot (WB) to visualize typical exosomal markers. The colloidal properties of the preparations were then evaluated by comparison with synthetic liposomes, nanoplasmonic colorimetric assay, Atomic Force Microscopy (AFM) and scanning Helium Ion Microscopy (HIM). Finally, the preparation’s biological activity was monitored by analyzing NfkB nuclear translocation induced in endothelial cells^24^. The work rationale is sketched in Fig. 1.

## Results and Discussion

### Separation from serum and biochemical characterization

**Figure 2:**
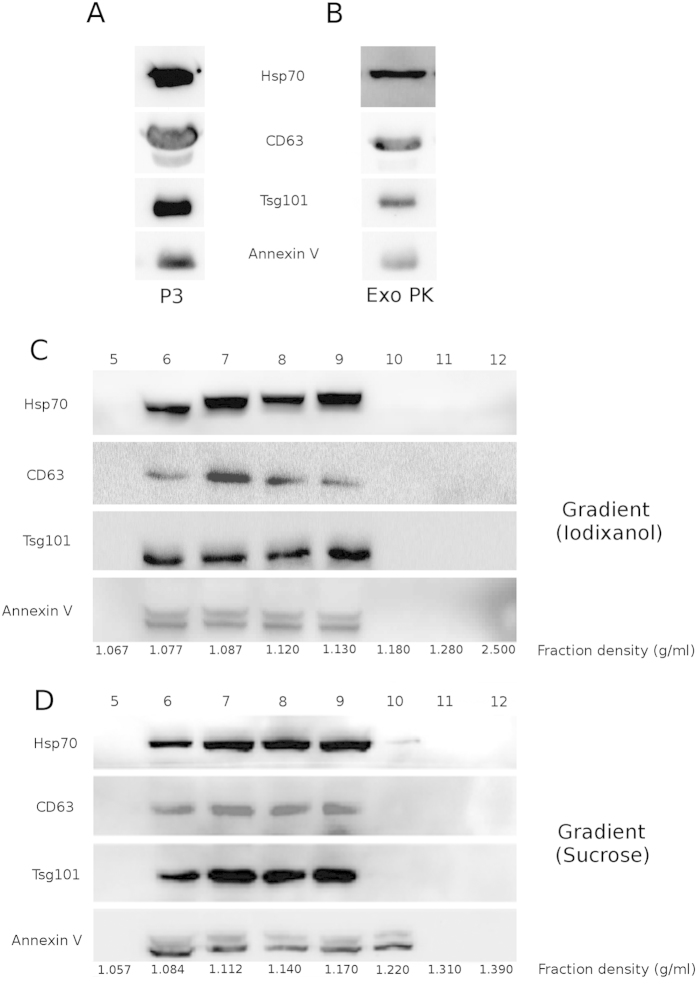
Biochemical characterization of exosome preparations: exosome preparations were obtained from 1 mL MM pool with four different protocols. Samples were electrophoresed and analyzed by Western Blot (WB) for the presence of typical vesicular markers. (**A**) Exosome preparation obtained with differential centrifugation steps (P3). (**B**) Exosome preparation purified with a precipitation kit (Exo PK). (**C**) Exosome preparation obtained with discontinuous iodixanol gradient. Top numbers refer to the corresponding gradient fraction. (**D**) Exosome preparation obtained with discontinuous sucrose gradient. Top numbers refer to the corresponding gradient fraction.

We isolated exosome populations from a pool of sera obtained from 20 patients with Multiple Myeloma (MM pool), which are very rich in extracellular vesicles in comparison with healthy donors^25^. Exosomes from serial centrifugation (P3) and the precipitation kit (Exo PK) were characterized by Western Blot (WB) for the presence of typical exosomal markers: the membrane fusion protein Annexin V, the tetraspannin CD63, the heat shock protein Hsp70 and TSG101, a protein which is involved in multivesicular body biogenesis^1^,^2^,^26^. Results are displayed (Fig. 2A,B). P3 protein concentration was (6 ± 1) μg/μL as quantified by Bradford assay.

P3 was subsequently loaded on top of two different discontinuous density gradients: iodixanol and sucrose. Twelve fractions were collected from each gradient and exosomal proteins were visualized by WB in fractions from 6 to 9 in both gradients. The biomarkers were detected in the range of 1.077–1.17 g/mL, as expected^27^ (Fig. 2C,D). Overall, these data indicate that, according to biochemical parameters conventionally adopted to verify the presence of exosomes in preparations, we obtained comparable and consistent exosomes populations with all the four different isolation protocols.

### Colloidal properties and purity

The first investigation on the colloidal properties and purity of the exosome preparations were conducted by comparing the preparations versus synthetic phosphatidylcholine (PCh) liposomes and Bovine Serum Albumin (BSA) on agarose gel runs. PCh liposome solutions can be exploited as a pure synthetic mimic of exosomes^28^,^29^. In this case, we prepared a control solution containing liposomes of 50–150 nm at 33 μM concentration and a BSA buffer solution (1 μg/μL) was used as additional control (see Methods for details). The membranous components of the preparations were labeled with the green fluorescent dye PKH67, while Coomassie Brilliant Blue (Coomassie) staining was used to reveal the whole protein content of the preparation, which is made of the proteins associated with the exosomes and of the single and aggregated exogenous protein contaminants.

**Figure 3:**
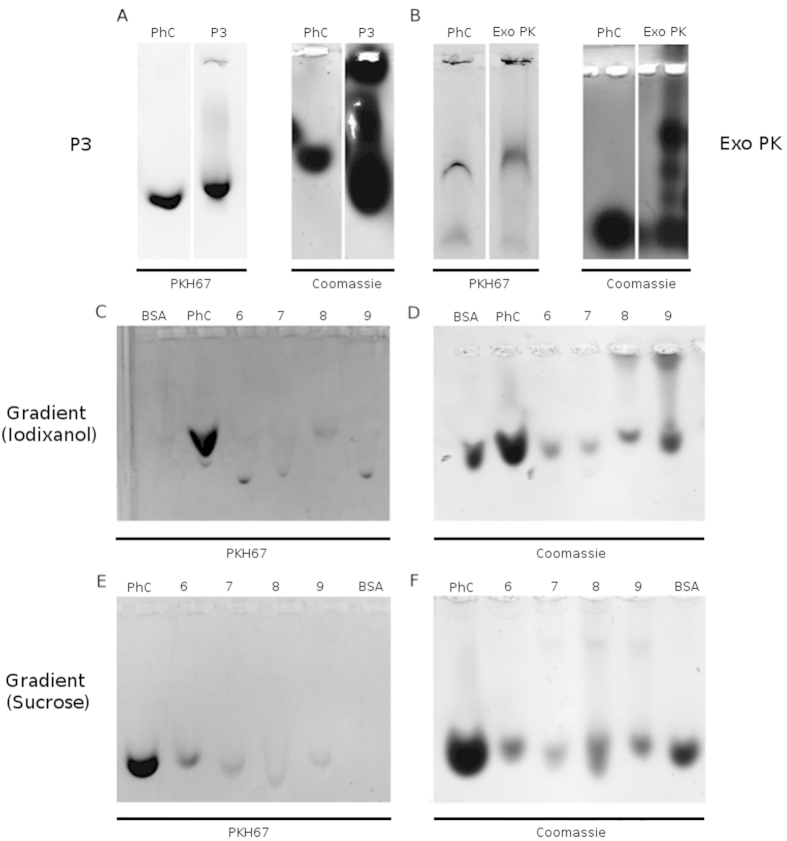
Residual matrix visualization: exosome preparations were isolated from 1 mL of MM pool with different protocols. Phosphatidylcholine liposomes (PhC, 33 μM) and vesicles were then stained with green fluorescent dye PKH67 and run on a 0.6% agarose gel. Gels were also stained with Coomassie Brilliant Blue dye. (**A**) PKH67 and Coomassie staining of PhC (10 μL) and exosome preparation obtained with serial centrifugation steps (P3). (**B**) PKH67 and Coomassie staining of PhC (10 μL) and exosome preparation obtained with a Exo PK. (**C**,**D**) PKH67 and Coomassie staining of Bovine serum albumin (BSA, 10 μg), PhC (10 μL) and exosomes preparation obtained with iodixanol gradient (Fractions 6–9). (**E**,**F**) PKH67 and Coomassie staining of BSA (10 μg), PhC (10 μL) and exosome preparation obtained with sucrose gradient (Fractions 6–9).

As displayed in Fig. 3 all the exosome preparations show the same migration pattern of PCh, confirming that they mainly contain lipid vesicles with a size typical of exosomes. This information well agrees with the AFM and HIM images reported and discussed later on. However, the Coomassie staining reveals a marked difference between the samples. Both P3 and Exo PK preparations show a pronounced dark smear, witnessing the presence of exogenous protein contaminants (Fig. 3A,B, right panels). The contaminants signal is instead absent in the Coomassie staining of fractions 6 and 7 of iodixanol and sucrose gradients (Fig. 3D,F).

Results on the purity of the preparations were confirmed by a nanoplasmonic colorimetric assay^28^. The assay exploits the property of a colloidal solution of gold nanoparticles (AuNPs) to turn from red to blue proportionally with the purity grade of the added exosome preparation. The color change is visible by eye and can be quantified by UV/vis/NIR spectroscopy. Briefly, bare AuNPs adsorb and cluster at the exosome membrane in pure preparations; instead, in the case of preparations containing exogenous contaminants, AuNPs are preferentially coated by those contaminants, which prevent AuNPs from clustering at the exosome membrane. Clustering drives a redshift of the localized surface plasmon resonance (LSPR) absorption peak of the AuNPs, i.e. a marked decrease of the absorption peak at 500–530 nm and an increase of the absorption peak at 650 nm^30^.

**Figure 4:**
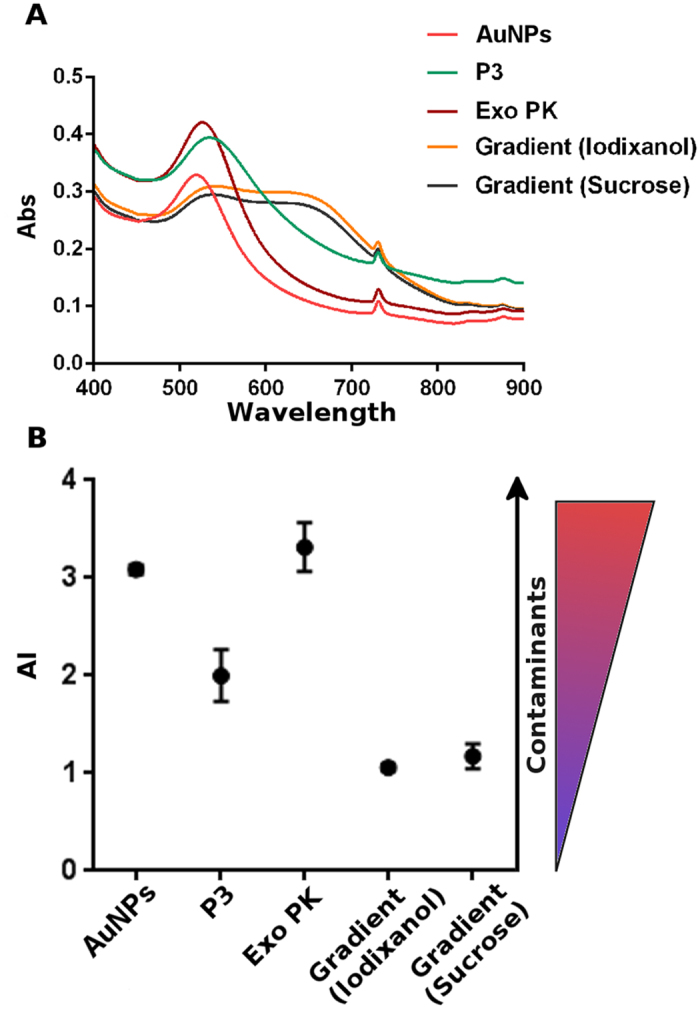
Biophysical samples’ purity grade determination. (**A**) Representative UV/vis/NIR spectra related to the colorimetric nanoplasmonic assay. Spectra were acquired with 1 nm step size from 400 nm to 900 nm wavelengths, (Abs, absorbance). (**B**) Aggregation index (*AI*) of the exosome preparations obtained from data as in panel A. *AI* was calculated as the ratio between the 519 nm and 650 nm LSPR absorbance. *AI*, decreses along with the change of the solution color from red to blue, and is inversely proportional to the preparation purity.

The UV/vis/NIR spectra of the assay reported in Fig. 4A show that the addition of the P3 and Exo PK preparations to the AuNP solution does not cause a significant shift of the LSPR peak of the pure AuNP solution, while the addition of the gradient preparations causes a marked redshift of the peak. This trend is more clearly evidenced by the plot of the aggregation index, *AI*, which directly relates the AuNP aggregation and the preparation purity grade (Fig. 4B) – *AI*, has been here conveniently defined as the ratio between the 519 nm and 650 nm LSPR absorbance (*AI* = *A*_519_/*A*_650_)^28^,^30^. – These assay responses translate the fact that the gradient preparations contain a lower amount of contaminants with respect to the P3 and Exo PK ones, in agreement with the results from the gel data (Fig. 3).

### AFM and HIM imaging

**Figure 5:**
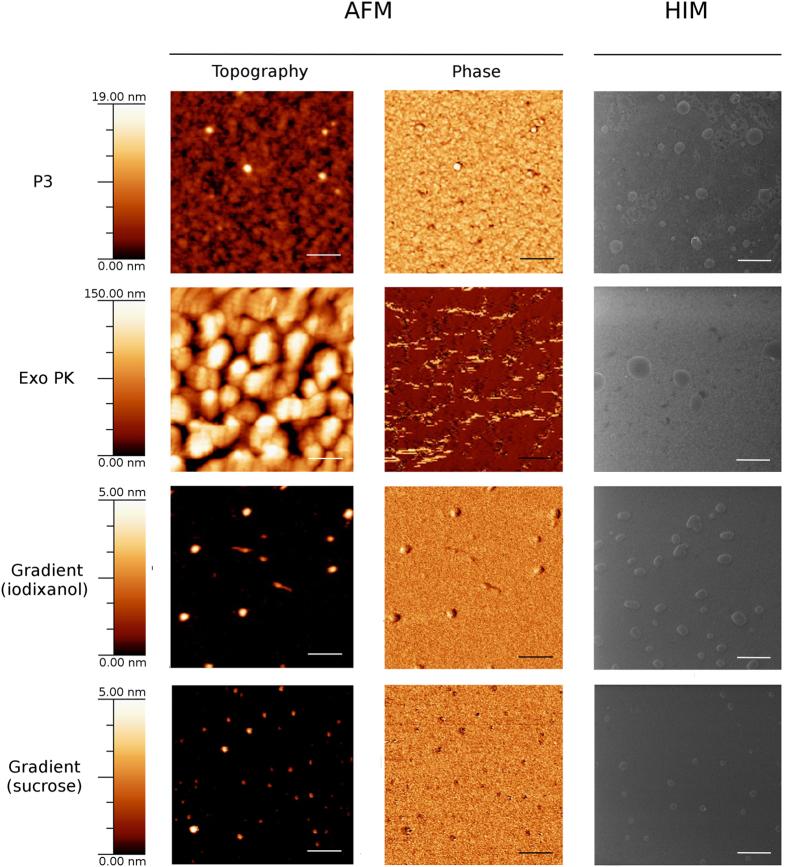
Imaging of exosome preparations: exosome preparations were obtained from 1 mL MM pool with four different protocols. Samples were examined by AFM (topography and phase mode) and HIM in order to visualize vesicle populations and the eventual residual matrix. Scale bars are 300 nm for the AFM pictures and 500 nm for the HIM pictures.

The microstructure of the exosome preparations was imaged by Atomic Force Microscopy (AFM) and scanning Helium Ion Microscopy (HIM). Representative images are reported in Fig. 5 and confirm that preparations are composed of vesicle populations of typical exosome size, consistently with exosome AFM and electron microscopy images reported in the literature^1^,^24^,^28^,^31^,^32^,^33^. The slight shape alteration from spherical to ellipsoidal in HIM images is due to the dehydration protocol used to fix the samples^34^.

The AFM topography image of the P3 preparation shows exosomes with size ranging from 50 to 100 nm. The topography and phase AFM images confirm the presence of a residual matrix, which surrounds the exosomes. The residual matrix, visible in the background, appears to be made of self-assembled small particles, which are not homogeneously adsorbed onto the mica surface (Fig. 5, P3). In addition, the AFM phase image shows that the P3 matrix and the exosomes have similar mechanical properties, suggesting that the matrix is made of biomolecules, such as residual aggregated serum proteins, mRNA enclosed inside portions of highly dense lipid membrane and insoluble immune-complexes. The HIM image allows for higher resolution and indicates that, in P3 sample, exosomes are very likely embedded into the matrix. In fact, in the P3 HIM image the exosomes display an unexpected surface roughness, which can be attributed to unspecific coating of proteins aggregates from the matrix. Also, the matrix can be clearly seen to underlie the exosomes forming island-like clusters.

Samples obtained with the Exo PK display a thicker and globular matrix that incorporates and/or covers the majority of the exosomes, compromising their visualization via AFM both in topography and phase modes. We believe that, in this case, the globular matrix is composed by the polymers used to precipitate exosomes and proteins. Via HIM the globular matrix shows a more compact pattern in respect to the one observed in the P3 sample. However, exosomes are visible, despite the fact that only low contrast images could be acquired, suggesting that matrix and exosomes have similar elemental composition and thickness. In agreement with what suggested by the colloidal characterization, also the Exo PK exosome preparations present a rough surface. Both the surface roughness and the low contrast images corroborate the picture of exosomes incorporated in a thick matrix.

In contrast, consistently with the colloidal characterization, the preparations from iodixanol and sucrose gradient do not present any trace of residual matrix. In addition, the HIM images evidence a smoother exosome surface. In these preparations the exosome population obtained by the sucrose gradient looks more monodispersed in size, with an average value of about 100 nm, with respect to the population from the iodixanol gradient, which spans a larger size range varying from 50 nm to 200 nm.

### Biological activity

Exosome biological activity can be influenced by their origin and conservation status^17^. Endothelial cell exposure to MM exosomes induce a pro-inflammatory environment in which the transcriptional factor NfkB is involved. We previously showed^24^ that HVEC (Human Vein Endothelial Cells) cells incubated with exosome preparations, isolated with sucrose gradient from serum of MM patients, internalize them. This event triggers a strong NfkB nuclear translocation. Here we exploit this property to investigate if the four exosome preparations characterized by different purity grades have different ability to induce NfkB nuclear translocation.

HVEC cells were incubated 4 h at 37 °C with preparations obtained with P3, Exo PK, iodixanol gradient, sucrose gradient or starvation buffer (RPMI 1640 without supplements); the latter used as control. Afterwards, we performed an immunofluorescence (IF) assay to visualize NfkB intracellular distribution. We quantified nuclear NfkB fluorescent signal, in comparison with the cytosolic signal then ratio between nuclear/cytosolic fluorescent signals was calculated.

**Figure 6:**
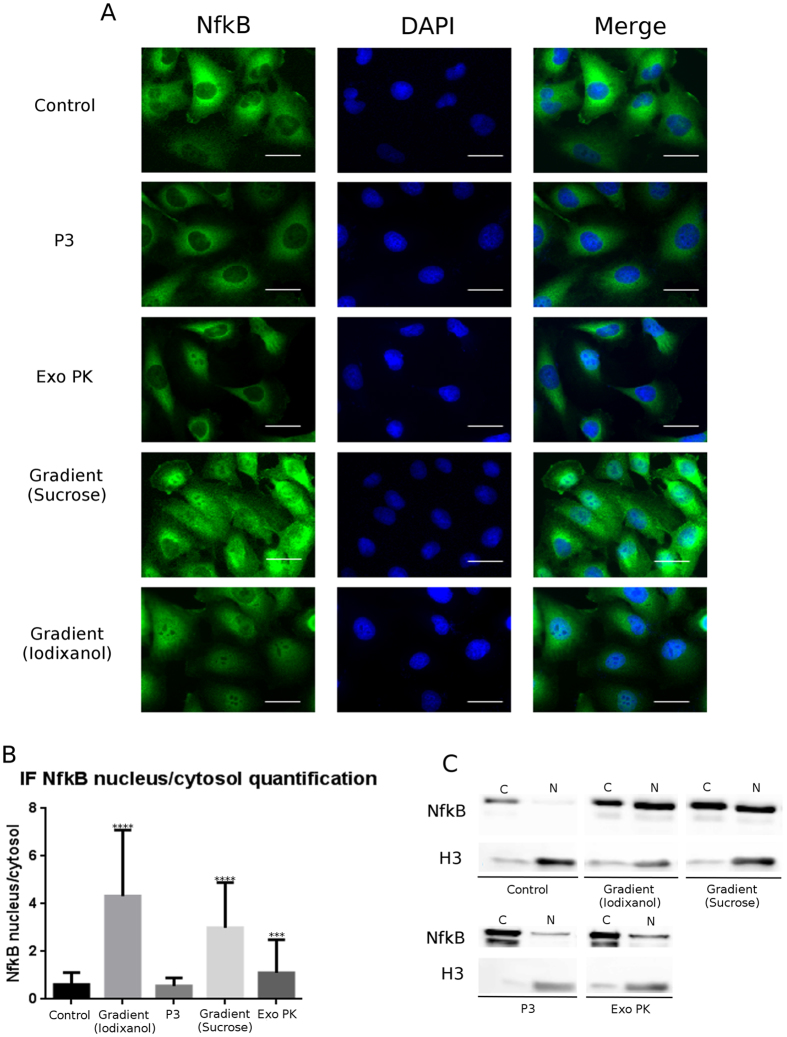
Residual matrix influences exosome preparations biological activity. (**A**) HVEC were incubated with exosome preparation deriving from 1 mL MM pool obtained with four different protocols for 4 h at 37 °C; as negative control (control) cells were starved in RPMI medium without supplements for 4 h. Single sections are shown for each condition. (**B**) Nuclear NfkB/Cytosolic NfkB fluorescent signal ratio. Fluorescence intensity measurement (100 cells each experimental point) was processed with the use of Image j. Mean value and ± SEM of three independent duplicate experiments are given. Significant differences in samples were determined with Student *t*-test (***p < 0.001 ****p < 0.0001) using Graph Pad program. (**C**) HVEC were treated as described and nuclei were separated from the cytosolic fraction by ultracentrifugation. Both nuclear and cytosolic fractions were electrophoresed and analyzed by WB using anti-NfkB and anti-Histone H3 antibodies.

From the sample images reported in Fig. 6 we learn that cells incubated with the P3 preparation showed no significant differences with respect to those treated with the control buffer; cells treated with the Exo PK preparation showed a faint NfkB nuclear signal. In stark contrast, cells incubated with the pure exosome preparations obtained from iodixanol and sucrose gradient, showed a clear strong NfkB nuclear translocation signal (Fig. 6A,B). These data were also confirmed by WB analysis of nuclear extracts reported in Fig. 6C, where an intense NfkB signal in the nuclei of cells incubated with gradient preparations is evident.

The origin of these results is probably related to the two steps mechanism, which determines the interaction between exosomes and target cells. The initial step is binding of exosomes to the target cell surface through ligand-receptor recognition and lipid-mediated interaction^27^. Numerous membrane-associated proteins decorate exosomes membranes and are functionally active on target cells^35^,^36^, driving exosome binding within minutes^37^,^38^. In the second step the attached exosomes are internalized by endocytosis pathways, which differ depending upon the recipient cell type^39^. In view of this mechanism and of the fact that in the contaminated samples the exosomes are embedded or surrounded by an exogenous matrix, we can reasonably infer that in the P3 and Exo PK preparations the residual matrix interferes/hampers the interaction between exosomes and cell membranes, and in turn their internalization and activation of NfkB translocation. Alternatively (or in concomitance) it is possible that residual matrix contains proteins that can block NfkB activation, particularly in P3 preparation.

To our knowledge, this is the first report describing that biological activity of exosome preparations from MM patient serum is influenced by residual contaminants, which may escape the purification procedure. The presence of contaminants cannot be assessed with standard biochemical assays used for exosomes but needs to be integrated with proper biophysical analysis. Indeed, there is currently no consensus on a “gold-standard” method to isolate and/or purify exosomes, probably because it is hard to identify an optimal universal method that is effective for any fluid^40^. It is of capital importance to highlight that the choice of exosomes isolation method from serum and, potentially, from other body fluids is essential. Protocols must be optimized to remove non-relevant proteins and sample purity grade should be checked and quantified at both the molecular and colloidal length scales, because it can strongly influence the final biological activity of exosome preparations.

## Methods

### Ethic Statement and sample collection

Serum samples of 20 patients with Multiple Myeloma (MM) were collected in the Laboratory of Clinical Biochemistry, Azienda Ospedaliera Spedali Civili of Brescia (AOSCB). After routine analysis, waste serum samples were coded, anonymized and frozen at −80 °C. The institutional review board of AOSCB approved the study in adherence with the Declaration of Helsinki. Informed consent was obtained from all subjects. All traceable identifiers were removed before analysis to protect patient confidentiality and all samples were analyzed anonymously. Patient’s serum were thawed, pooled together (MM pool) and subsequently analyzed as described in the next sections. All experiments were performed in accordance with the approved guidelines. The institutional review board of Azienda Ospedaliera Spedali Civili of Brescia approved this study (REC number: SFLC01).

### Separation by serial centrifugation steps (P3)

One mL serum sample from MM pool was processed with serial centrifugation steps as previously described^23^ (800 × g for 30 min, 16,000 × g for 45 min, 100,000 × g for 2 h). Final pellet is hereafter referred as P3. To quantify P3 protein concentration pellet was re-suspended in 100 μL 10 mM Tris–HCl, pH 7.4 and analyzed by Bradford assay.

### Separation by discontinuous sucrose gradient

P3 was further processed as previously reported^24^. Briefly, P3 was re-suspended in 800 μL buffer A (10 mM Tris–HCl 250 mM sucrose, pH 7.4), loaded on top of a discontinuous sucrose gradient (15, 20, 25, 30, 40, 60% sucrose in 10 mm Tris–HCl, pH 7.4) and centrifuged at 100,000 × *g* for 16 h at 4 °C (rotor MLS 50, Beckman Optima MAX). Twelve fractions with equal volumes (400 μL) were collected from the top of the gradient, and vesicles were pelleted by ultracentrifugation (100,000 × g for 2 h). Pellets were then re-suspended in 50 μL of 100 mM Tris, 150 mM NaCl, 1 mM EDTA supplemented with 1:1000 Protease Inhibitor Cocktail (P.I., Sigma) and analyzed as described. Western Blot (WB) fractions containing exosome markers (from 6 to 9, with a density of 1.077–1.13 g/mL) were ultracentrifuged at 100,000 × g for 1 h and further analyzed.

### Separation by discontinuous iodixanol gradient

P3 was also processed by discontinuous iodixanol gradient, as previously reported^24^. Briefly, P3 was re-suspended in 80 μL buffer A (10 mM Tris–HCl 250 mM sucrose, pH 7.4). To prepare the discontinuous iodixanol gradient, 40% (w/v), 20% (w/v), 10% (w/v) and 5% (w/v) solutions of iodixanol were made by diluting a stock solution of OptiPrep™ (60% (w/v) aqueous iodixanol from Axis-Shield PoC) with 10 mM Tris, pH 7.4. The gradient was formed by adding 1.2 mL of 40% iodixanol solution followed by careful layering of 1.2 mL each of 20% and 10% solutions, and 1.12 mL of 5% solution^20^. Centrifugation was performed at 100,000 × g for 16 h at 4 °C. Twelve fractions with equal volumes (400 μL) were collected from the top of the gradient, and vesicles were pelleted by ultracentrifugation (100,000 × g for 1 h). The pellets were analyzed as described in the text.

### Separation by precipitation kit (Exo PK)

Exosomes from 1 mL serum sample were isolated using the “Total Exosome Isolation (from serum)” kit (Invitrogen) according to manufacturer’s instructions. The reagent contained in the kit forces less-soluble components, such as vesicles, out of solution, allowing them to be collected by a short, low-speed centrifugation (10,000 × g for 10 min). Pellet was re-suspended as described in each experiment and analyzed as described in the text.

### Nanoplasmonic colorimetric assay

Exosome preparations were checked for purity by adapting the colorimetric nanoplasmonic assay we developed. The assay exploits the properties of the solutions containing gold nanoparticles (AuNPs) and exosomes. For further details on the assay working principle see Ref. 28 and the Results and Discussion Section.

The different exosome preparations were treated for being used in the colorimetric assay as it follows. Fractions 6–9 from iodixanol were collected together and centrifuged 2 h at 100,000 × g. Same protocol was applied for sucrose gradient fractions. P3, Exo PK and gradients fractions were re-suspended in 100 μL 0.9% NaCl, diluted 1:16 with deionazed H_2_O and mixed with a final concentration of 3 nM gold nanoparticles (AuNPs, 15 nm), The blue shift was quantified by collecting the UV-Vis spectra of the different AuNP-exosome solutions. UV-Vis spectra were measured with a JASCO UV-Vis spectrophotometer and acquired with 1 nm step size in a wavelength window ranging from 400 nm to 900 nm. The AuNPs aggregation index (AI) was defined as the ratio of the LSPR absorption at 519 and 650 nm (AI = A519/A650).

### Phosphatidylcholine liposomes

Synthetic phosphatidylcholine (PCh) liposomes were prepared as previously described^28^. Briefly for PCh liposomes preparation the proper amount of (1-palmitoyl–2-oleoyl-sn-glycero-3-phosphocholine, POPC, purchased from Avanti Lipids) was dissolved in chloroform/methanol 6:1 (v/v). A lipid film was obtained by evaporating the solvent under a stream of nitrogen and overnight vacuum drying. The film was then swollen and suspended in warm (50 °C) 0.9% NaCl solution by vigorous vortex mixing. To prepare vesicles with narrow distribution, the dispersion was tip-sonicated for 30 minutes as described in ref. 28. The number of vesicles was evaluated as described in Maiolo *et al*.^28^.

### Fluorescent labeling

Liposomes, P3, Exo PK and fractions from different gradients were fluorescently labeled as described previously^24^. Briefly preparations were re-suspended in Diluent C (PKH67 Green Fluorescent cell linker, Sigma) to a 70 μL final volume. 1,7 μL of PKH67 green fluorescent dye was added to each sample and incubated at room temperature for 10 min. The reaction was stopped adding 70 μL of 1% BSA in PBS 1X. Exosome preparations were centrifuged at 100,000 × g for 2 h.

### Vesicle and protein detection on agarose gel

Liposomes, P3, Exo PK and fractions from different gradients were labeled with PKH67 membrane dye as described above. Samples were loaded on a 0.6% agarose gel and electrophoresed 30 min at 100 V as previously reported^28^. Fluorescent signal was acquired using a G:Box Chemi XT Imaging system (Syngene). The gel was, then, stained with Coomassie Brilliant Blue for 1 h at 37 °C and destained with Destaining solution (10% Acetic acid, 20% Methanol). Images were acquired using a G:Box Chemi XT Imaging system (Syngene).

### Atomic Force Microscopy (AFM) imaging

Exosome preparations obtained with different purification protocols were re-suspended in 50 μL of 100 mM Tris, 150 mM NaCl, 1 mM EDTA and diluted 1:10 with deionized water. Five to 10 μL of samples were then spotted onto freshly cleaved mica sheets (Grade V-1, thickness 0.15 mm, size 15 × 15 mm). All mica substrates were dried at room temperature and analyzed using a JEOL JSPM-4210, equipped with Veeco or MikroMasch AFM tips. Images were snapped in tapping mode; scan size ranged from 0.3 to 15 μm and scan speed ranged from 0.6 to 3.3 ms x clock^41^.

### Scanning Helium Ion Microscopy (HIM) imaging

Exosome preparations obtained from different protocols were re-suspended in 20 μL of 100 mM Tris, 150 mM NaCl, 1 mM EDTA and diluted 1:10 with milli-Q water. Each sample was adsorbed on carbon coated copper grids and fixed with modified Karnovsky’s Mixture (2% Paraformaldehyde, 2.5% Glutaraldehyde and 0.1 M Sodium Phosphate Buffer). Hence, adsorbed exosomes were dehydrated with ethanol 99% and dried in a critical point drying device. All samples were imaged using a helium ion microscope (HIM, Orion Plus™, Carl Zeiss located at Consorzio GRINT) equipped with an Everhart-Thornley detector. Images were acquired in secondary electron mode with an acceleration voltage of 30 kV with a probe current ranging from 0.3 to 1.0 pA. Neutrality of the samples was maintained through the use of a low energy electron flood gun properly synchronized with the imaging beam.

### Cell Culture

HVEC (Human Vein Endothelial Cells) cells were grown in RPMI 1640 (Euroclone) supplemented with 10% Fetal bovine serum (FBS) (Lonza), 1% Penicillin/Streptomycin (Lonza), 1% Glutamine (Lonza), at 37 °C, 5% CO_2_.

### NfkB nuclear translocation induction

Gradient fractions 6–9 were collected and spun at 100,000 × g for 2 h. P3, Exo PK and gradients (iodixanol and sucrose) pellets were re-suspended in 200 μL RPMI 1640 without supplements (starvation medium). HVEC monolayer was washed two times with PBS 1X and incubated with 100 μL starvation medium (Control) or with 100 μL of re-suspended exosomes preparation for 4 h at 37 °C. Cells were then analyzed for immunofluorescence (IF) or WB analysis.

### Fractionation of cytosolic, membranous and nuclear components

HVEC cell monolayers were treated as described above. Cells were then processed as previously described in Di Noto *et al*.^24^.

### Immunofluorescence (IF) imaging

HVEC cells were cultured on 35 mm glass coverslips until 60–80% of confluency and incubated at 37 °C with exosome preparations or starvation medium as described above. Cells were then treated as previously described (Di Noto *et al*.^24^, nuclear staining). Primary antibody: anti-NfkB, (Santa-Cruz). Secondary antibodies: Alexa Fluor 488 and DAPI. Fluorescent microscopy was performed on a ZEISS Axiovert 100 fluorescent microscope using a 63x oil immersion objective. NfkB nuclear and cytoplasmic fluorescence intensity quantification was determined using Image J on at least 100 cells for each treatment. Background was subtracted to nucleus and cytosol signal and ratio between Nuclear/Cytosolic fluorescent signal was calculated.

### Antibodies and Immunoblotting

The following antibodies were used in our experiments: mouse anti-Hsp70 (Enzo Life Science), rabbit anti-NfkB (Santa-Cruz), mouse anti-CD63 (Millipore), mouse anti-Tsg 101 (Abcam), mouse anti-Annexin V (Santa-Cruz), and goat anti Histone H3 (Santa Cruz).

Exosome preparations from MM pool were obtained as described before. Samples were normalized for protein concentration (Bradford Assay) when possible (in alternative equal volumes of each sample were loaded on a acrylamide–bisacrylamide gel), boiled in reducing SDS sample buffer (80 mM Tris, pH 6.8, 2% SDS, 7.5% glycerol, 0.01% Bromophenol blue) supplemented with 2% 2-mercaptoethanol (Sigma) for 5 min at 95 °C and separated by SDS–PAGE on a acrylamide/bisacrylamide (10% or 12.5%) gel and analyzed by WB as previously described^24^.

### Statistical analysis

Significant differences among control datasets and other samples were determined with Student’s *t*-test (Graph Pad). *P* values of less than 0.05 were considered statistically significant with ***p < 0.001 and ****p < 0.0001. Values were shown as mean ± Standard Error of the mean (SEM) of at least 3 experiments.

## Additional Information

**How to cite this article**: Paolini, L. *et al*. Residual matrix from different separation techniques impacts exosome biological activity. *Sci. Rep.* **6**, 23550; doi: 10.1038/srep23550 (2016).

## Figures and Tables

**Figure 1:**
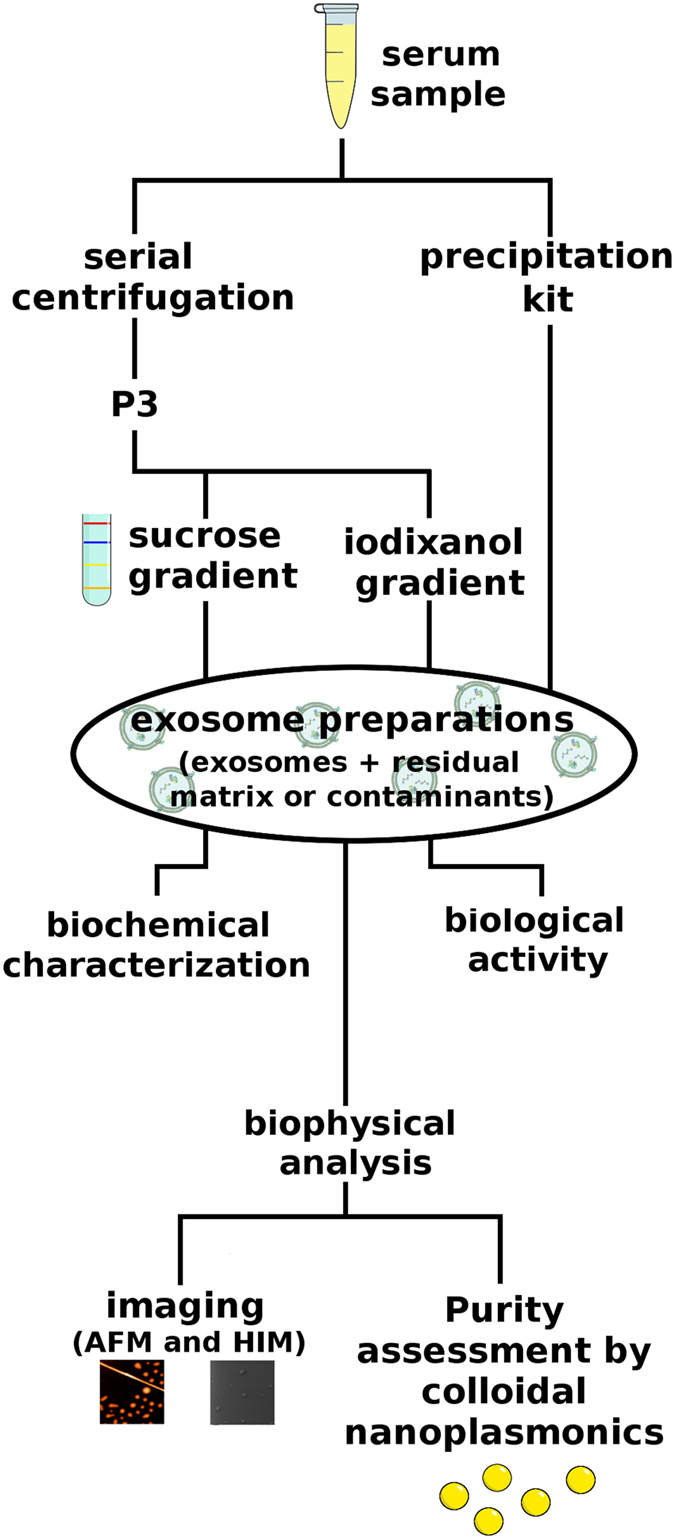
Residual matrix from different separation techniques impacts exosome preparations’ biological activity: rationale sketched.
